# Incidence of recurrent ischemic stroke and its associated factors in a tertiary care center in Thailand: a retrospective cohort study

**DOI:** 10.1186/s12883-024-03640-0

**Published:** 2024-05-04

**Authors:** Thanapoom Taweephol, Pitsinee Saksit, Akarin Hiransuthikul, Pongpat Vorasayan, Wasan Akarathanawat, Aurauma Chutinet

**Affiliations:** 1 Department of Microbiology, Faculty of Medicine, Chulalongkorn University, Bangkok, 10330 Thailand; 2 Faculty of Medicine, Chulalongkorn University, Bangkok, 10330 Thailand; 3 Department of Preventive and Social Medicine, Faculty of Medicine, Chulalongkorn University, Bangkok, 10330 Thailand; 4 Division of Neurology, Department of Medicine, Faculty of Medicine, Chulalongkorn University, Bangkok, 10330 Thailand; 5 Chulalongkorn Stroke Center, Chula Neuroscience Center, King Chulalongkorn Memorial Hospital, Thai Red Cross Society, Bangkok, 10330 Thailand

**Keywords:** Recurrent, Ischemic stroke, Incidence rate, Risk factors, Log-rank test

## Abstract

**Background:**

Ischemic stroke (IS) is one of the leading causes of death among non-communicable diseases in Thailand. Patients who have survived an IS are at an increased risk of developing recurrent IS, which can result in worse outcomes and post-stroke complications.

**Objectives:**

The study aimed to investigate the incidence of recurrent IS among patients with first-ever IS during a one-year follow-up period and to determine its associated risk factors.

**Methods:**

Adult patients (aged ≥ 18 years) who were hospitalized at the Stroke Center, King Chulalongkorn Memorial Hospital (KCMH) in Bangkok, Thailand, due to first-ever IS between January and December 2019 and had at least one follow-up visit during the one-year follow-up period were included in this retrospective cohort study. IS diagnosis was confirmed by neurologists and imaging. The log-rank test was used to determine the event-free survival probabilities of recurrent IS in each risk factor.

**Results:**

Of 418 patients hospitalized due to first-ever IS in 2019, 366 (87.6%) were included in the analysis. During a total of 327.2 person-years of follow-up, 25 (6.8%) patients developed recurrent IS, accounting for an incidence rate of 7.7 per 100 person-year (95% confidence interval [CI] 5.2–11.3). The median (interquartile range) time of recurrence was 35 (16–73) days. None of the 47 patients with atrial fibrillation developed recurrent IS. The highest incidence rate of recurrent IS occurred within 1 month after the first episode (34 per 100 person-years) compared to other follow-up periods. Patients with small vessel occlusion and large-artery atherosclerosis (LAA) constituted the majority of patients in the recurrent IS episode (48% and 40%, respectively), with LAA exhibiting a higher recurrence rate (13.5%). Additionally, smoking status was found to be associated with an increased risk of recurrence.

**Conclusion:**

The incidence rate of the recurrence was moderate in our tertiary care setting, with a decreasing trend over time after the first episode. The various subtypes of IS and smoking status can lead to differences in event-free survival probabilities.

## Introduction

Stroke is one of the major causes of morbidity and mortality worldwide, especially in low- and middle-income countries [1]. In Thailand, stroke is the leading cause of death among non-communicable diseases and carries the highest disease burden measured by disability-adjusted life years (DALYs) in both male and female ≥ 60 years [2]. Major risk factors for stroke in Thai population include hypertension, diabetes, dyslipidemia, metabolic syndrome, smoking, and atrial fibrillation. Ischemic stroke (IS) is the most common type of stroke in Thailand, with lacunar infarction being the most common subtype [3]. Unfortunately, the prevalence of stroke among Thai population has doubled from 1.12 to 2.56% within a decade [4, 5].

Patients who survive on the first episode of IS are at increased risk of recurrence, with approximately half of recurrences occurring within the first few days and weeks after the initial event [6, 7]. A recent study, employing a meta-analysis approach, revealed that the annual risk of recurrent stroke was 4.26% across 34 studies. Further analysis demonstrated that the annual risk of fatal recurrent stroke stood at 0.77% based on 18 studies, while the annual risk of non-fatal recurrent stroke was determined to be 2.92% from 20 studies [8]. Besides, the recurrence rates persist at relatively moderate to high levels based on findings from several past studies. The one-year recurrence rate, documented in five studies conducted in China, Korea, Japan, and Spain, ranges from 5.7 to 17.7%, encompassing diverse populations and study types [9–13]. Despite advancements in guidelines for secondary prevention and the implementation of antiplatelet/antithrombotic treatments for strokes, recurrence rates exhibit notable variations among different populations. This variability underscores the importance of investigating recurrence rates and comprehending their association with risk factors.

Several risk factors have been identified for recurrent IS, including increasing age, hypertension, diabetes mellitus, hyperlipidemia, obesity, and cardiovascular diseases such as atrial fibrillation, ischemic heart disease, and cardiomyopathy. Lifestyle factors such as a history of smoking, sleep deprivation, poor dietary intake, and insufficient physical exercise have also been linked to an increased risk of recurrence [14, 15]. Furthermore, patients with a prior history of IS with high National Institutes of Health Stroke Scale (NIHSS) and high Modified Rankin Scale (mRS) scores are at an increased risk of the recurrence [14]. Recurrent IS also worsens functional outcomes, increases disability and institutionalization, and raises in-hospital costs and mortality rates [16–19]. Therefore, it is important for healthcare providers to identify early those patients who are at high risk of developing recurrent IS.

The aims of this study were to investigate the incidence of the recurrent IS (the second episode) in one-year-period follow-up among adult patients with first-ever IS and to determine the risk factors associated with recurrence.

## Methods

### Study design

A retrospective cohort study was conducted in the stroke unit, Chulalongkorn Stroke Center of Excellence, KCMH. All patients older than 18 years admitted with the first-ever IS in the stroke unit, between January 1st and December 31st, 2019, were included in this study. All follow-ups within one-year period after their discharge were reviewed. Therefore, patients who came to follow-up visit at least one time were included in the analysis.

IS was defined as an episode of neurological deficit lasting more than 24 h with clinical symptoms based on pathological or imaging evidence of infarction related to the clinical findings. The definition of recurrent IS in our study was a new neurological deficit manifesting after the first episode of IS that was clinically stable, lasting more than 24 h, and readmitted to the stroke unit, KCMH. The imaging evidence is confirmed by neuroradiologists or experienced stroke neurologists. The Trial of Org 10,172 in Acute Stroke Treatment (TOAST) classification was used for subtypes of IS based on etiology and clinical subtypes were classified with the Oxfordshire Community Stroke Project (OCSP) classification. Clinical characteristics, NIHSS [20], mRS [21], comorbidities, and medical history were registered during an admission.

### Statistical analysis

Demographic data was summarized as frequency and percentage for categorical variables, and median with interquartile range (IQR) for continuous variables. Comparisons between groups (those with and without recurrent IS) were performed using Pearson’s chi-squared, Fischer’s exact, and Mann-Whitney U test, and comparisons between episodes (those with the first and second episode of IS) were performed using marginal homogeneity and Wilcoxon sign rank test as appropriate. The primary outcome was incidence of recurrent IS and its associated risk factors. The log-rank test was used to assess the event-free survival probabilities of recurrent IS in each risk factor. Patients without recurrent IS events were censored at the last day they were known to be alive within the 1-year follow-up period or at the end of the 1-year follow-up period. Statistical significance was defined as *p*-value of < 0.05. All statistical analyses were performed using the Stata/SE, version 17.0 (StataCorp LP, College Station, TX).

## Results

Between January 1 and December 31, 2019, 418 patients with first-ever IS were admitted to KCMH. A total of 366 (87.6%) patients had at least one follow-up visit and were included in the analysis. The median age (IQR) was 65 (55–75) years, 192 (52.5%) were female, 265 (72.4%) had hypertension, 126 (34.4%) had diabetes mellitus, 177 (48.4%) had dyslipidemia, 47 (12.8%) had atrial fibrillation, and the median (IQR) NIHSS score at admission was 4 (2-7). According to TOAST classification, 187 (51.1%) were small vessel occlusion (SVO), 74 (20.2%) were large-artery atherosclerosis (LAA), and 68 (18.6%) were cardioembolism (CE). Lacunar infarct (LACI) found in 199 (54.4%) patients was the most common subtype from OCSP classification. Compared to patients who did not develop recurrent IS, those who developed recurrent IS were less likely to have dyslipidemia (28.0% vs. 49.9%, *p*-value = 0.035) and more likely to smoke (40.0% vs. 21.4%, *p*-value = 0.032). None of the 47 patients with atrial fibrillation developed recurrent IS. Their anticoagulant treatment consisted of 31 patients receiving warfarin, 1 patient receiving enoxaparin, 7 patients receiving apixaban, 1 patient receiving edoxaban, and 2 patients receiving rivaroxaban (Table 1).

**Table 1:** Baseline characteristics of patients with and without recurrent ischemic stroke

Characteristics	Total (*n* = 366)	Non-recurrent (*n* = 341)	Recurrent (*n* = 25)	*p*-value
Age (years), median (IQR)	65 (55–75)	65 (56–76)	64 (48–73)	0.156
Female	192 (52.5)	181 (53.1)	11 (44.0)	0.38
TOAST classification				0.006
LAA	74 (20.2)	64 (18.8)	10 (40.0)	
CE	68 (18.6)	68 (19.9)	0 (0)	
SVO	187 (51.1)	175 (51.3)	12 (48.0)	
OD	12 (3.3)	10 (2.9)	2 (8.0)	
UD	25 (6.8)	24 (7.0)	1 (4.0)	
OCSP classification				0.662
TACI	13 (3.6)	13 (3.8)	0 (0)	
PACI	121 (33.1)	111 (32.6)	10 (40.0)	
LACI	199 (54.4)	187 (54.8)	12 (48.0)	
POCI	33 (9.0)	30 (8.8)	3 (12.0)	
NIHSS score on admission, median (IQR)	4 (2–7)	4 (2–7)	4 (3–5)	0.543
Treatment				
IV thrombolysis	72 (19.7)	69 (20.2)	3 (12.0)	0.437
Endovascular thrombectomy	20 (5.5)	20 (5.9)	0 (0)	0.381
Hypertension	265 (72.4)	248 (72.7)	17 (68.0)	0.61
Diabetes mellitus	126 (34.4)	117 (34.3)	9 (36.0)	0.864
Dyslipidemia	177 (48.4)	170 (49.9)	7 (28.0)	0.035
Obesity	124 (33.9)	116 (34.0)	8 (32.0)	0.837
Atrial fibrillation	47 (12.8)	47 (13.8)	0 (0)	0.057
Prior myocardial infarction	34 (9.3)	33 (9.7)	1 (4.0)	0.493
Obstructive sleep apnea	2 (0.6)	2 (0.6)	0 (0)	> 0.999
Valvular heart disease	16 (4.4)	15 (4.4)	1 (4.0)	> 0.999
Peripheral arterial disease	3 (0.8)	2 (0.6)	1 (4.0)	0.192
Smoking	83 (22.7)	73 (21.4)	10 (40.0)	0.032
Alcohol	42 (11.5)	37 (10.9)	5 (20.0)	0.187
Time to hospital (hours), median (IQR)	7 (3–24)	7 (3–24)	9 (4–18)	0.75
Systolic blood pressure (mmHg), median (IQR)	159 (140–176)	160 (140–178)	149 (140–168)	0.175
Diastolic blood pressure (mmHg), median (IQR)	87.5 (77–99)	87 (77–99)	89 (84–94)	0.523
Length of stay (days), median (IQR)	4 (3–7)	4 (3–7)	4 (3–5)	0.356
NIHSS score at discharge, median (IQR)	2 (0–4)	1 (0–4)	3 (0–4)	0.731
mRS at discharge, median (IQR)	1 (1–2)	1 (1–2)	1 (0–3)	0.432

Abbreviations: CE: cardioembolism; IQR: interquartile range; IV: intravenous; LAA: large artery atherosclerosis; LACI: lacunar infarct; mRS: modified Rankin Scale; NIHSS: National Institutes of Health Stroke Scale; OCSP: the Oxfordshire Community Stroke Project Classification; OD: stroke of other determined etiology; PACI: partial anterior circulation infarct; POCI: posterior circulation infarct; SVO: small vessel occlusion; TACI: total anterior circulation infarct; TOAST: the Trial of Org 10,172 in Acute Stroke Treatment; UD: stroke of undetermined etiology

From an overall follow-up time of 327.2 person-years, 25 (6.8%) developed recurrent IS with an incidence rate of 7.7 per 100 person-years (95% confidence interval [CI] 5.2–11.3) and a median (IQR) time of recurrence of 35 (16–73) days. The incidence rate within 1 month after the first episode was higher (34.0 per 100 person-years) than the incidence rates within 3 months (24.3 per 100 person-years) and 12 months (7.7 per 100 person-years) (Table 2). In subgroup comparisons, there were no significant differences between age, sex, and mRS at discharge. According to TOAST classification, there was a statistically significant difference in event-free survival probabilities of those incidence rates within 12 months (*p*-value = 0.01), whereas, corresponding to OCSP classification, there was no difference in event-free survival probabilities within any range of follow-up time. Increased event-free survival probabilities within 3 months were found in those patients with an NIHSS score greater than 5 on both admission and discharge (*p*-value = 0.043 and 0.04, respectively). In concordance with Table 1, patients who had dyslipidemia had increased event-free survival probabilities within 1 month and 12 months (*p*-value = 0.025 and 0.03, respectively) and those who had history of smoking had decreased event-free survival probabilities within 12 months (*p*-value = 0.036) (Table 2).

**Table 2:** Incidence rate of recurrent ischemic stroke per 100 person-years

Demographic variables	Numbers of events within 1 month	Incidence rate within 1 month (95% CI)	*p*-value	Numbers of events within 3 months	Incidence rate within 3 months (95% CI)	*p*-value	Numbers of events within 12 months	Incidence rate within 12 months (95% CI)	*p*-value
Overall	10	34.0 (18.3–63.1)	NA	21	24.3 (15.9–37.3)	NA	25	7.7 (5.2–11.3)	NA
Age group			0.106			0.768			0.288
<60 years	6	59.5 (26.7-132.4)		10	34.0 (18.3–63.2)		11	10.3 (5.7–18.5)	
≥60 years	4	20.7 (7.8–55.0)		11	19.3 (10.7–34.9)		14	6.4 (3.8–10.8)	
Sex			0.568			0.333			0.401
Female	6	39.2 (17.6–87.3)		9	19.8 (10.3–38.1)		11	6.5 (3.6–11.7)	
Male	4	28.3 (10.6–75.3)		12	29.4 (16.7–51.7)		14	9.0 (5.3–15.1)	
TOAST classification			0.792			0.231			0.01
LAA	2	33.6 (8.4-134.3)		9	53.3 (27.7-102.4)		10	17.5 (9.4–32.5)	
CE	0	0		0	0		0	0	
SVO	7	46.7 (22.3–97.9)		10	22.7 (12.2–42.1)		12	7.0 (4.0-12.3)	
OD	1	115.5 (16.3–820.0)		1	39.9 (5.6-282.9)		2	26.0 (6.5-104.2)	
UD	0	0		1	16.6 (2.3-118.1)		1	4.2 (0.6–29.5)	
OCSP classification			0.864			0.514			0.632
TACI	0	0		0	0		0	0	
PACI	2	20.4 (5.1–81.6)		8	28.1 (14.1–56.2)		10	9.6 (5.1–17.8)	
LACI	6	37.5 (16.8–83.4)		10	21.2 (11.4–39.4)		12	6.6 (3.8–11.6)	
POCI	2	76.1 (19.0-304.4)		3	38.8 (12.5-120.4)		3	10.4 (3.3–32.1)	
NIHSS score on admission			0.749			0.043			0.29
0–5	9	46.4 (24.1–89.2)		16	28.3 (17.3–46.1)		19	8.7 (5.6–13.7)	
>5	1	9.9 (1.4–70.6)		5	16.9 (7.0-40.5)		6	5.5 (2.5–12.3)	
Hypertension			0.653			0.841			0.603
Yes	6	28.1 (12.6–62.6)		14	22.4 (13.3–37.8)		17	7.1 (4.4–11.4)	
No	4	49.3 (18.5-131.5)		7	29.5 (14.1–61.9)		8	9.2 (4.6–18.3)	
Diabetes mellitus			0.677			0.446			0.894
Yes	2	19.5 (4.9–77.8)		7	23.3 (11.1–48.9)		9	8.0 (4.2–15.4)	
No	8	41.7 (20.9–83.4)		14	24.9 (14.7–42.0)		16	7.5 (4.6–12.2)	
Dyslipidemia			0.025			0.295			0.03
Yes	1	6.9 (1.0-49.1)		5	11.7 (4.9–28.0)		7	4.2 (2.0-8.9)	
No	9	60.0 (31.2-115.4)		16	36.9 (22.6–60.2)		18	11.1 (7.0-17.7)	
Obesity			0.441			0.926			0.818
Yes	3	30.0 (9.7–93.0)		5	17.0 (7.1–40.8)		8	7.0 (3.5–14.1)	
No	7	36.0 (17.2–75.5)		16	28.2 (17.2–45.9)		17	8.0 (5.0-12.8)	
Smoking			0.677			0.201			0.036
Yes	2	29.7 (7.4-118.6)		9	46.4 (24.2–89.3)		10	13.9 (7.5–25.9)	
No	8	35.2 (17.6–70.4)		12	17.9 (10.2–31.6)		15	5.9 (3.6–9.8)	
Alcohol			0.979			0.274			0.162
Yes	3	90.2 (29.1-279.7)		4	41.7 (15.7-111.2)		5	13.7 (5.7–32.8)	
No	7	26.8 (12.8–56.2)		17	22.2 (13.8–35.7)		20	6.9 (4.5–10.7)	
NIHSS score at discharge			0.509			0.04			0.3
0–5	10	40.3 (21.7–74.8)		19	26.1 (16.7–41.0)		23	8.3 (5.5–12.4)	
>5	0	0		2	14.7 (3.7–58.9)		2	4.12 (1.0-16.5)	
mRS at discharge			0.858			0.984			0.205
0–2	7	30.4 (14.5–63.9)		13	19.2 (11.2–33.1)		17	6.6 (4.1–10.6)	
3–6	3	46.4 (15.0-144.0)		8	42.7 (21.4–85.4)		8	11.8 (5.9–23.5)	

Abbreviations: CE: cardioembolism; CI: confidence interval; IQR: interquartile range; LAA: large artery atherosclerosis; LACI: lacunar infarct; mRS: modified Rankin Scale; NA: not available; NIHSS: National Institutes of Health Stroke Scale; OCSP: the Oxfordshire Community Stroke Project Classification; OD: stroke of other determined etiology; PACI: partial anterior circulation infarct; POCI: posterior circulation infarct; SVO: small vessel occlusion; TACI: total anterior circulation infarct; TOAST: the Trial of Org 10,172 in Acute Stroke Treatment; UD: stroke of undetermined etiology

Of 25 patients who developed recurrent IS, evaluating the first and second episode, there were no differences in TOAST and OCSP classification, together with NIHSS score on admission, time to hospital, NIHSS score and mRS at discharge, length of stay, systolic blood pressure, and diastolic blood pressure. In addition, 7 out of 10 (70%) patients who had LAA and 8 out of 12 (66.7%) patients who had SVO developed the same TOAST classification at both episodes; besides, 9 out of 10 (90%) patients who had PACI and 9 out of 12 (75%) who had LACI established the same OCSP classification (Table 3).

**Table 3:** Characteristics of first and second episode of ischemic stroke

Characteristics	First episode (*n* = 25)	Recurrence (*n* = 25)	*p*-value
TOAST classification			0.954
LAA	10 (40.0)	10 (40.0)	
CE	0 (0)	0 (0)	
SVO	12 (48.0)	11 (44.0)	
OD	2 (8.0)	2 (8.0)	
UD	1 (4.0)	2 (8.0)	
OCSP classification			0.223
TACI	0 (0)	0 (0)	
PACI	10 (40.0)	12 (48.0)	
LACI	12 (48.0)	12 (48.0)	
POCI	3 (12.0)	1 (4.0)	
NIHSS score on admission, median (IQR)	4 (3–5)	4 (2–6)	0.935
Time to hospital (hours), median (IQR)	9 (4–18)	13 (3–72)	0.158
Systolic blood pressure (mmHg), median (IQR)	149 (140–168)	150 (123–167)	0.777
Diastolic blood pressure (mmHg), median (IQR)	89 (84–94)	82 (78–100)	0.353
Length of stay (days), median (IQR)	4 (3–5)	3 (3–5)	0.946
NIHSS score at discharge, median (IQR)	3 (0–4)	3 (0–5)	0.214
mRS at discharge, median (IQR)	1 (0–3)	1 (1–3)	0.227

Abbreviations: CE: cardioembolism; IQR: interquartile range; LAA: large artery atherosclerosis; LACI: lacunar infarct; mRS: modified Rankin Scale; NIHSS: National Institutes of Health Stroke Scale; OCSP: the Oxfordshire Community Stroke Project Classification; OD: stroke of other determined etiology; PACI: partial anterior circulation infarct; POCI: posterior circulation infarct; SVO: small vessel occlusion; TACI: total anterior circulation infarct; TOAST: the Trial of Org 10,172 in Acute Stroke Treatment; UD: stroke of undetermined etiology

## Discussion

In this study, we estimated the incidence of recurrent IS. Most recurrences occurred in the first month, with median time to recurrence of around 1 month. Differences in survivor functions were associated with factors such as TOAST classification, NIHSS score, dyslipidemia, and history of smoking. Although there were no differences between the characteristics of patients in the first and second episodes, most of them developed the same IS subtypes.

The incidence rate of recurrent IS in this study was 7.7 per 100 person-years, which was relatively in the middle compared to earlier studies which have a broad range of the incidence rates for recurrent IS per 100 person-years at 1 year, including 0.1 in Taiwan, 2.5 in women and 3 in men in Sweden, 6.8 in Norway, 10.8 in Whites and 15.4 in Blacks in the United States [22–25]. The median time to recurrence of 35 days in this study falls within the range previously reported from Norway, Australia, and the United States, which is a moderately wide range of time from 1 to 255 days [26–28]. In this study, we also calculated the incidence rate at 1 month and 3 months, which gives us a robust trend to approximate that within the first month, in concordance with the median time to recurrence, after the first-ever IS, we should considerably prepare for secondary prevention of the recurrence. We hypothesize that the reasons for our relatively moderate outcomes are racial differences, income level, patient education, and access to treatment. First, while the Asian population has a lower risk of developing cerebrovascular diseases compared to non-Asians, only a few studies have reported on recurrent IS, and the results still vary [29–31]. Next, in upper-middle-to-high-income countries in Asia, the income level might play a role in the treatment outcomes from the first-ever IS and secondary prevention, which could lead to lower incidence or decreasing trends of recurrent IS [23, 32]. Lastly, if we closely examine the time taken to reach the hospital, the median from patients in this study was more than 4.5 h. This is an important consideration for intravenous thrombolytic therapy, which might indicate a lack of patient education or punctual access to medical management in our country.

Our study examined several baseline factors and their association with event-free survival probabilities. With regards to TOAST classification, differences in event-free survival probabilities within one year may reflect an association between IS subtypes and recurrence in our study. While there are few studies reporting on the incidence rates of recurrent IS classified by subtypes, those that do report varying results. Some studies suggest that LAA and CE subtypes have a higher recurrence rate within one year, while patients with SVO have a lower recurrence rate compared to others [10, 33–35]. Our study found that differences in the incidence rates of NIHSS scores at admission and discharge within 3 months were associated with increased event-free survival probabilities in patients with an NIHSS score greater than 5, which contradicts previous studies suggesting a correlation between higher NIHSS scores and recurrence [36–38]. However, there were no differences in other follow-up periods. The observed result in our study could be attributed to patients with an NIHSS score greater than 5 exhibiting a more disabled condition, potentially contributing to improved compliance with medication and other lifestyle modifications. Alternatively, patients with a higher NIHSS score may not experience a recurrent episode due to comorbidities and complications, leading to death before recurrence. Moreover, our findings in patients with atrial fibrillation were inconsistent with previous reports, which have indicated a strong association between atrial fibrillation and recurrence [10, 35]. One possible explanation for our results is that none of the patients with atrial fibrillation who developed a recurrent IS were part of the small number of index recurrent cases. Alternatively, it could be due to adequate treatment with anticoagulants and patient compliance in our hospital, which may have reduced the risk of recurrent IS.

Dyslipidemia was found to increase event-free survival probabilities within 1 month and 12 months. This is in contrast to earlier studies that associated dyslipidemia with a higher risk of recurrent IS and other cerebrovascular diseases [10, 39]. Following national recommendations on secondary prevention of IS, all patients received high-intensity statin therapy after the initial IS episode [40]. Therefore, we hypothesized that our findings could be attributed to the longer exposure to statin therapy in patients with dyslipidemia prior to their first-ever IS episode. Although, to our knowledge, there is no data on the effect of pre-stroke statin on the incidence of recurrent IS, studies have suggested potential benefits of early statin initiation, albeit from IS patients. A randomized controlled trial involving 6,100 patients in China demonstrated that immediate intensive statin therapy initiated after stroke onset significantly decreased the risk of poor functional outcome, although it was not found to reduce the risk of recurrent IS within 90 days compared to a three-day delayed intensive statin therapy [41]. In addition, a population-based study of up to 60,000 people in Finland showed that early statin initiation within 90 days of the IS episode significantly reduced the cumulative incidences of recurrent IS, as well as all-cause mortality and major adverse cardiovascular events, for up to at least 12 years compared to those who did not, [40] suggesting the benefits of early statin use. We also postulated that those who have had dyslipidemia and were taking pre-stroke statin may have been more adherent to their medications and committed to lifestyle modifications. Unfortunately, we lack the data on both the pre-stroke statin use and the medication adherence to support our hypothesis in our study. Additionally, there was a possibility that patients with dyslipidemia adhered more effectively to medications and committed to lifestyle modifications. Furthermore, we found decreased event-free survival probabilities within 12 months of follow-up in patients who smoke, which is in line with several previous studies [35, 37]. The understanding of the association between smoking and cerebrovascular diseases has evolved over time. Smoking generates oxidative stress and chronic inflammation, resulting in an endothelial dysfunction and an increased risk of IS [42, 43]. Accordingly, smoking has been found to increase the risk of recurrence [44]. Besides, these groups of patients who smoke demonstrate differences in characteristics from the beginning. It is necessary to consider this when interpreting the results, as these factors could contribute to explaining the outcomes in our study. Therefore, smoking cessation should be a priority for every patient who currently smokes or is at risk of smoking in the near future.

No significant differences were found in the characteristics of the episodes in the same patients. Most of the patients in our study experienced the same IS subtypes during recurrence, which is consistent with previous studies. Approximately half of the recurrent cases had the same subtypes or almost all subtypes from patients in the recurrent episode of IS were identical [35, 45, 46]. The similarity in subtypes observed in both the first and recurrent episodes could be attributed to their existing pathology, with the majority of patients exhibiting LAA and SVO subtypes. Moreover, when examining the time to hospital, although no significant difference was found, it is remarkable that patients in the recurrent group took more time to reach the hospital. The greatest time to hospital was 72 h, which may have clinical importance. This group of patients, having experienced an IS once, and their caregivers may have overlooked symptoms or assumed they were related to the initial lesion. This could result in a delayed presentation to the hospital, potentially leading to worse outcomes. Additionally, the median of NIHSS score at admission and discharge were equivalent between episodes. While some studies in the Asian population may lack this type of data, we accomplished demonstrating this information from Asian patients in a low-to-middle-income country.

Certain limitations need to be considered in our study. First, our study comprised a relatively small sample size, limiting our ability to perform additional extensive analyses, particularly in adjusting for potential confounding factors when establishing association between baseline factors and recurrent IS. Secondly, there is a limitation in the generalizability, especially to other ethnicities and hospital scales, as all study participants are Thai, and our study was conducted in a single tertiary care medical center in Thailand. Thirdly, due to the retrospective nature of the study design, the recurrence rate of IS in our study may be underestimated due to the possibility that some patients might not have been re-hospitalized at the same hospital for the recurrent episode. In addition, this also led to the unavailability of certain data, including information on pre-stroke statin use and medication adherence. Nevertheless, we successfully collected one-year data and provided time-related information, a data that is scarce in low-to-middle-income countries.

## Conclusion

We present epidemiological data on the incidence of recurrent IS in Thailand, with incidence rates observed in tertiary care settings. Our findings indicate that the incidence rate of recurrent IS in Thailand is relatively moderate compared to previous studies, with a decreasing incidence rate observed over time following a first-ever IS. Comprehensive secondary prevention strategies based on stroke subtypes should be implemented for every patient, particularly within the first month following the first-ever IS.

## Data Availability

We understand the importance of data access and transparency, and we strive to maintain the highest standards when it comes to handling and providing access to data. If you request specific data that falls within the scope of our data, we can provide it to assist you.
